# Vitiligo in a Patient With Kabuki Syndrome: Case Study and Review of the Literature

**DOI:** 10.7759/cureus.34143

**Published:** 2023-01-24

**Authors:** Karli Gage, Amanda S Weissman, Jeffrey McBride

**Affiliations:** 1 Department of Dermatology, OU-TU School of Community Medicine, Tulsa, USA; 2 Department of Dermatology, University of Oklahoma Health Sciences Center, Oklahoma City, USA

**Keywords:** janus kinase inhibitors, vitiligo, autoimmune disease, child, genetic diseases

## Abstract

Kabuki Syndrome (KS) is a rare genetic disorder characterized by dysmorphic facial features, skeletal anomalies, dermatoglyphic abnormalities, intellectual disability, and short stature. Autoimmune disease can be seen more frequently in this patient population. Vitiligo is an autoimmune disease that is uncommonly reported in patients with KS. This report describes a case of vitiligo manifesting in a patient with KS and discusses the use of Janus kinase inhibitors as treatment.

## Introduction

Kabuki Syndrome (KS), also known as Niikawa-Kuroki syndrome, is a rare genetic disorder characterized by dysmorphic facial features, skeletal anomalies, dermatoglyphic abnormalities, intellectual disability, and short stature [1]. The characteristic facial features of KS are reminiscent of the makeup used by actors in the traditional Japanese Kabuki theatre, hence the syndrome’s name [1]. KS is most commonly caused by mutations in lysine-specific methyltransferase 2D (KMT2D), occurring in 56-75% of individuals [2].

The estimated prevalence of KS varies between 1:32,000 births in Japan and 1:86,000 births in New Zealand and Australia [3]. Autoimmune disease, such as immune thrombocytopenic purpura (ITP), can be seen more frequently in this patient population [4]. Vitiligo, however, is not commonly reported in patients with KS [3]. This case report describes a patient with KS who is diagnosed with widespread nonsegmental vitiligo, reviews the literature on KS patients with vitiligo, and discusses a newer class of medication treating nonsegmental vitiligo with minimal adverse effects.

## Case presentation

**Figure 1:**
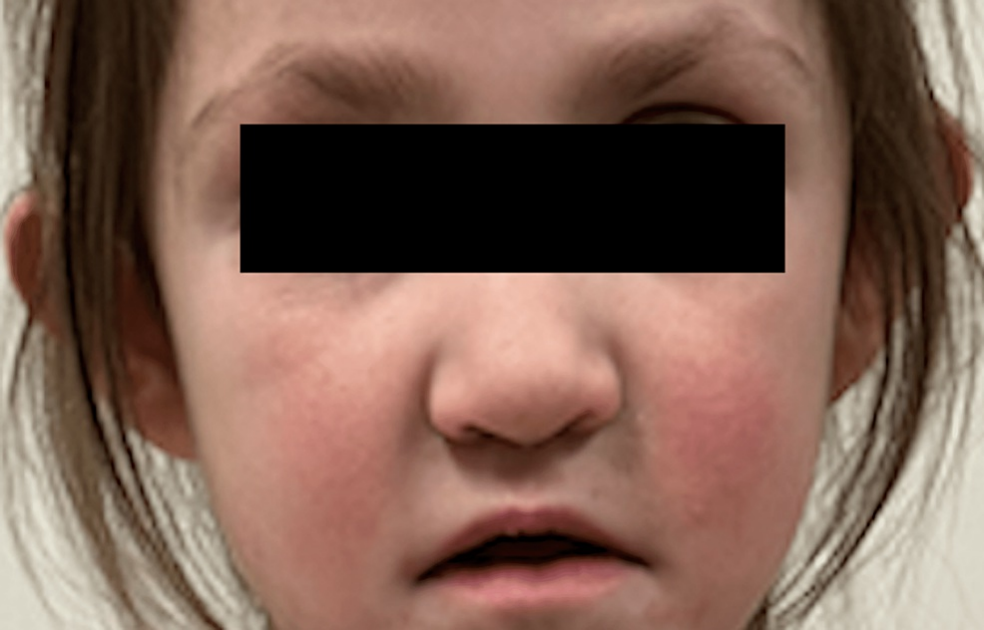
Characteristic dysmorphic facies of our patient with Kabuki Syndrome

A 5-year-old female presents to dermatology for a one-year duration of increasing, asymptomatic light patches on her skin. Further history reveals the patient has Kabuki Syndrome discovered by whole exome sequencing which found a pathogenic variant in the KMT2D gene (Table 1). This mutation has led to dysmorphic facial features (Figure 1), developmental delay, imperforate anus with recto-vestibular fistula, gastrointestinal malrotation, gastric reflux, feeding difficulties, pulmonary hypertension, coarctation of the aorta, left aortic arch with aberrant right subclavian artery, mitral stenosis, bicuspid aortic valve, ventricular septal defect, left hydronephrosis, right renal dysplasia, chronic otitis media, bilateral hearing loss, and congenital dislocation of the right hip.

**Table 1:** Results of patient's whole exome sequencing

Gene	Disease	Mode of Inheritance	Variant	Zygosity	Inherited From	Classification
KMT2D	Kabuki Syndrome	Autosomal Dominant	c.14189 G>A p.W4730X	Heterozygous	De Novo	Pathogenic Variant

**Figure 2:**
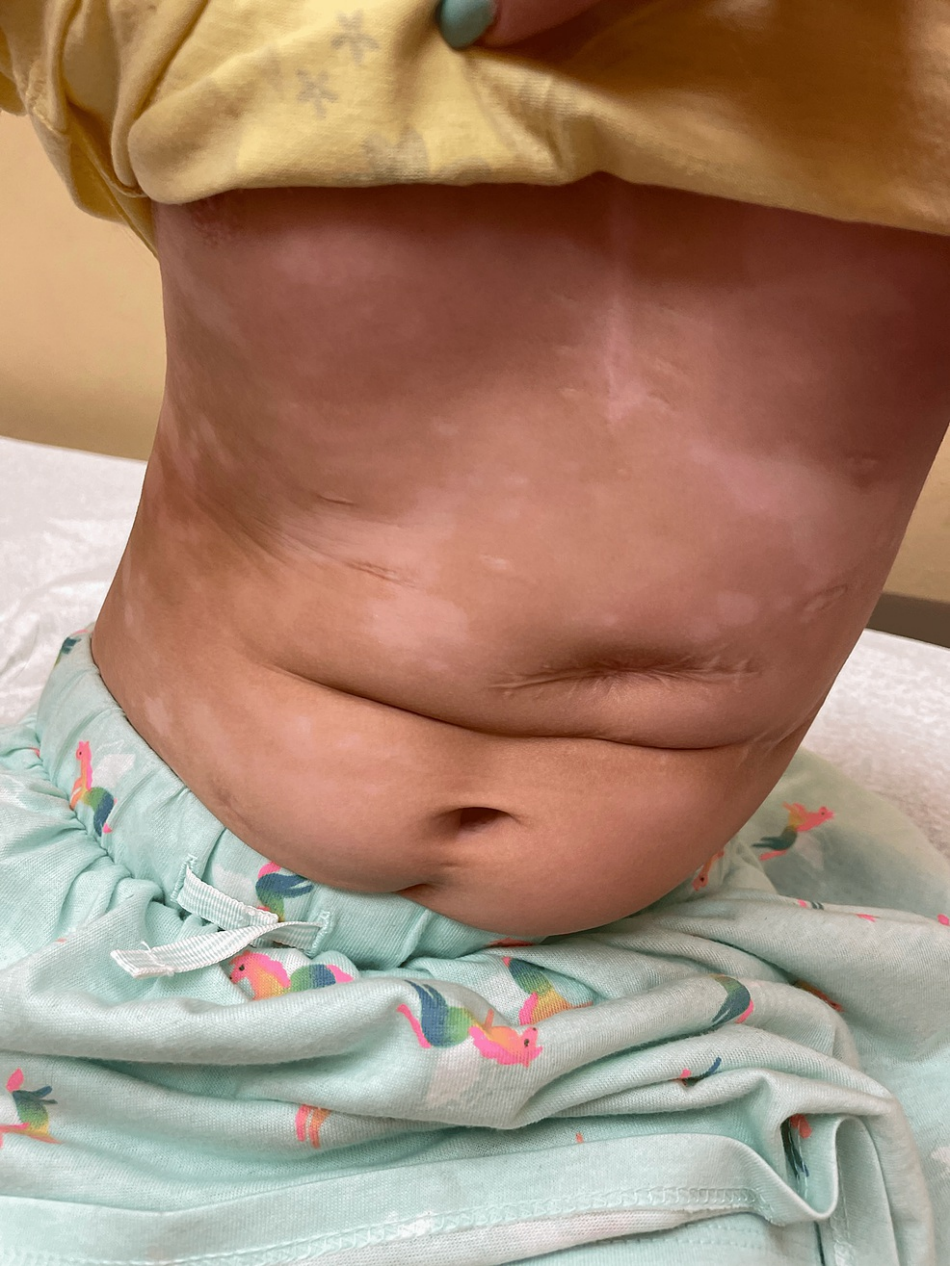
Depigmented macules and patches on the patient’s trunk

**Figure 3:**
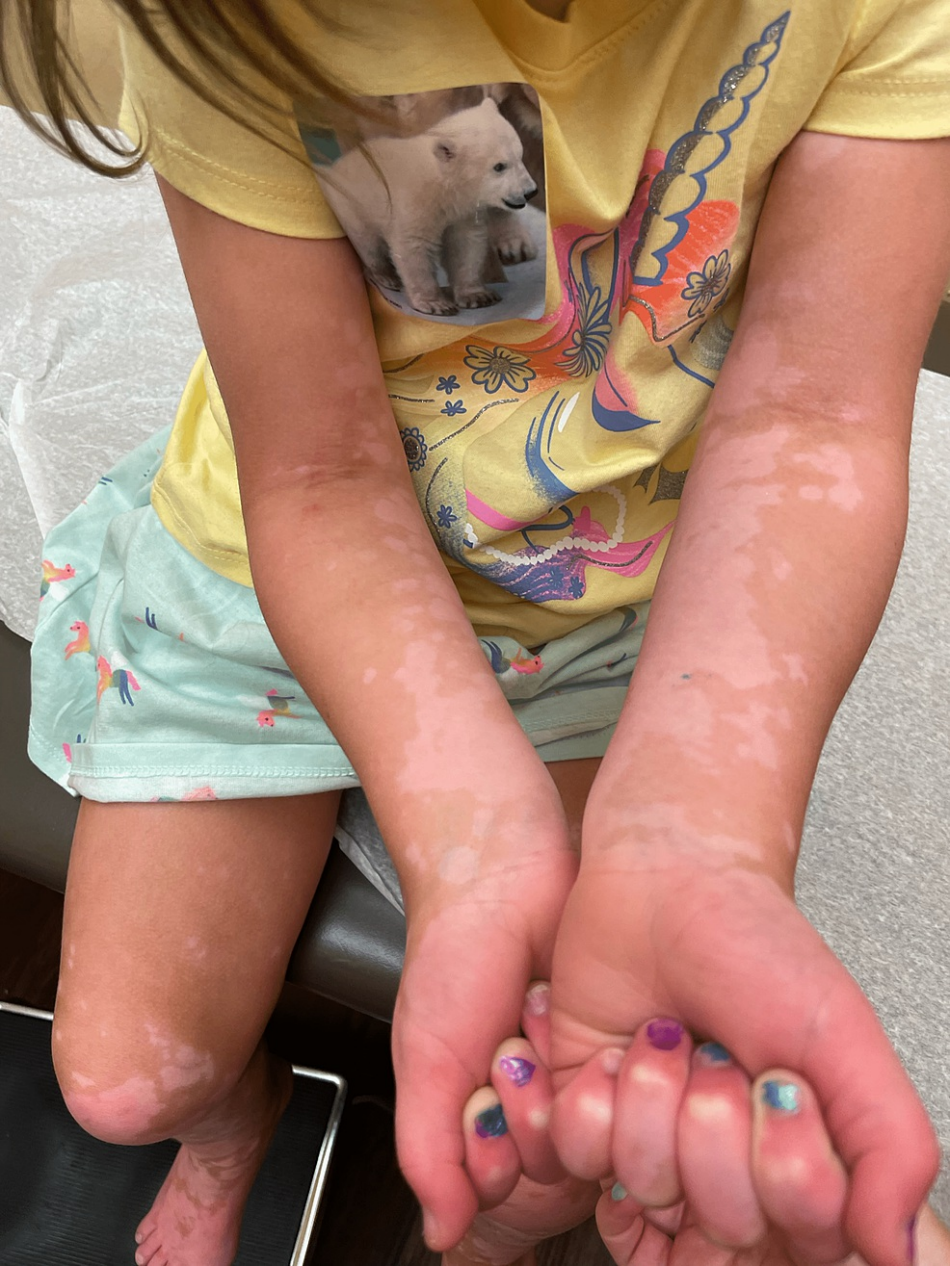
Depigmented macules and patches on the patient’s upper extremities

**Figure 4:**
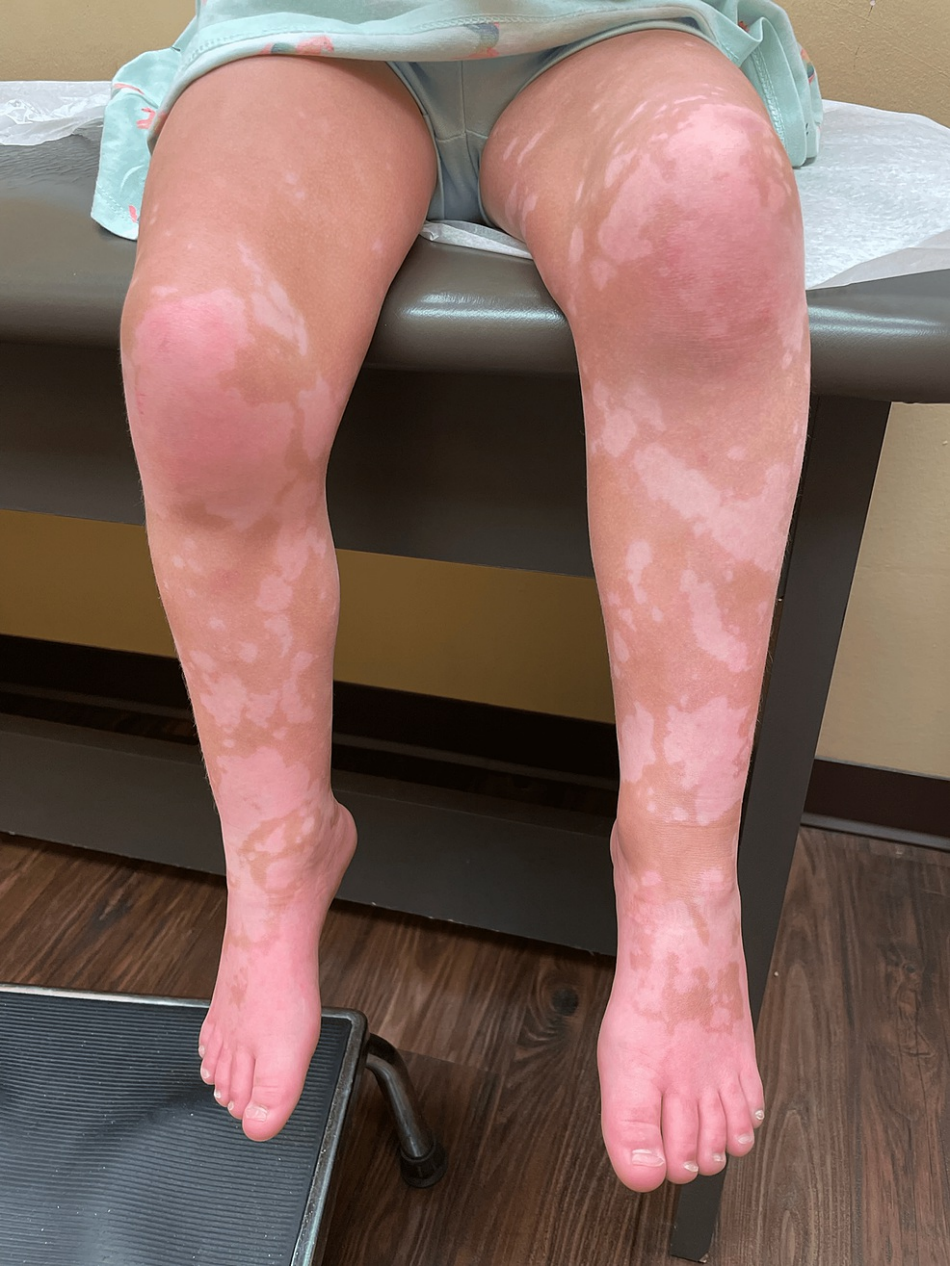
Depigmented macules and patches on the patient’s lower extremities

**Figure 5:**
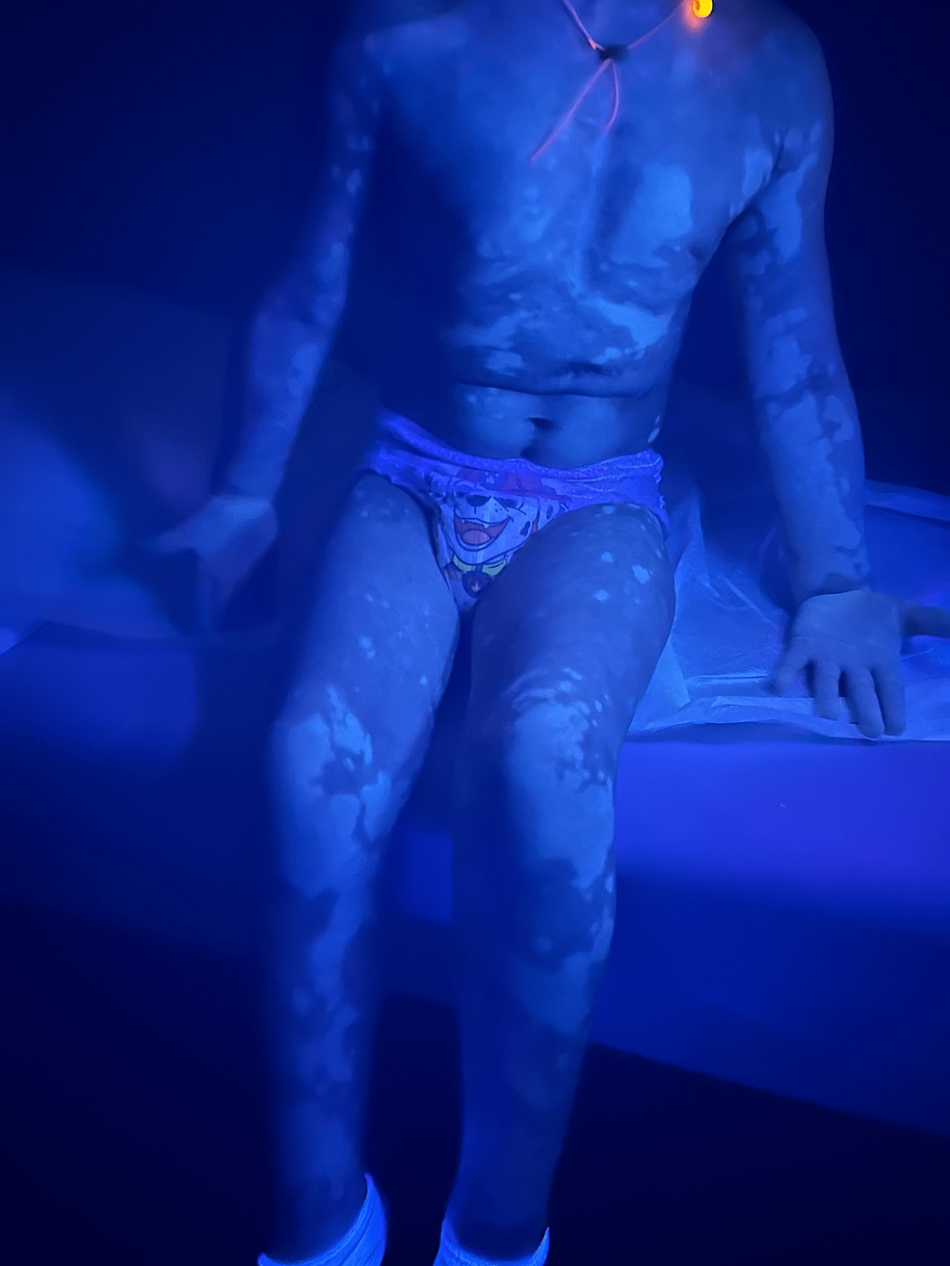
Accentuated depigmented macules and patches under Wood's lamp

Upon examination, there are many coalescing depigmented patches on the patient’s neck, trunk, and extremities (Figures 2-4). The face is spared. Under a Wood's lamp, the patches accentuate, revealing lesions with sharp borders and a bright blue-white fluorescence (Figure 5). A diagnosis of nonsegmental vitiligo was made, and the patient was started on tacrolimus 0.03% ointment to be applied to the affected areas twice daily. An additional treatment of topical 1.5% ruxolitinib applied twice daily to less than 10% of the body surface was initially prescribed; however, due to cost, the patient decided on sole treatment with tacrolimus.

**Figure 6:**
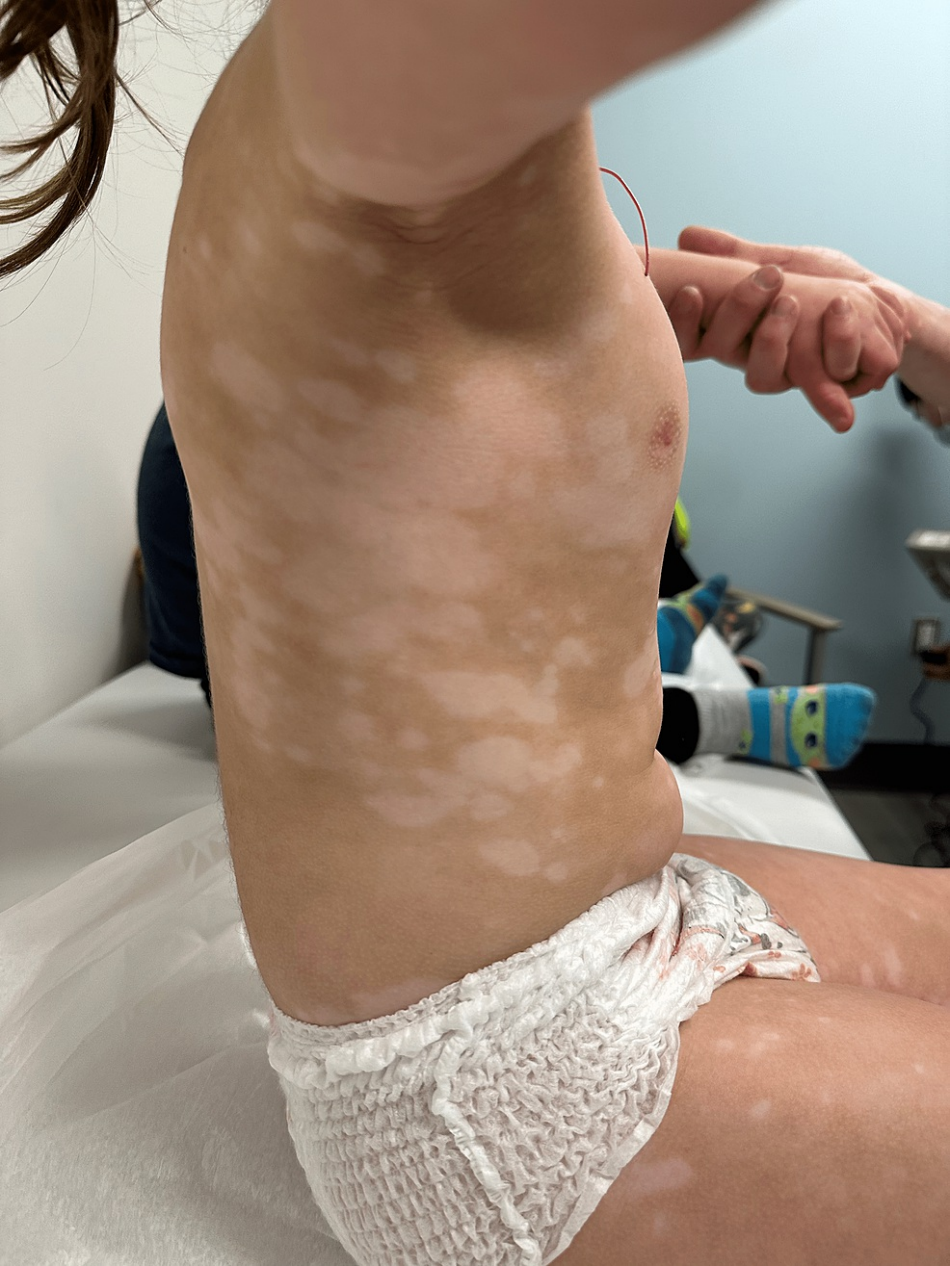
Depigmented macules and patches on the patient's trunk at four-month follow-up

**Figure 7:**
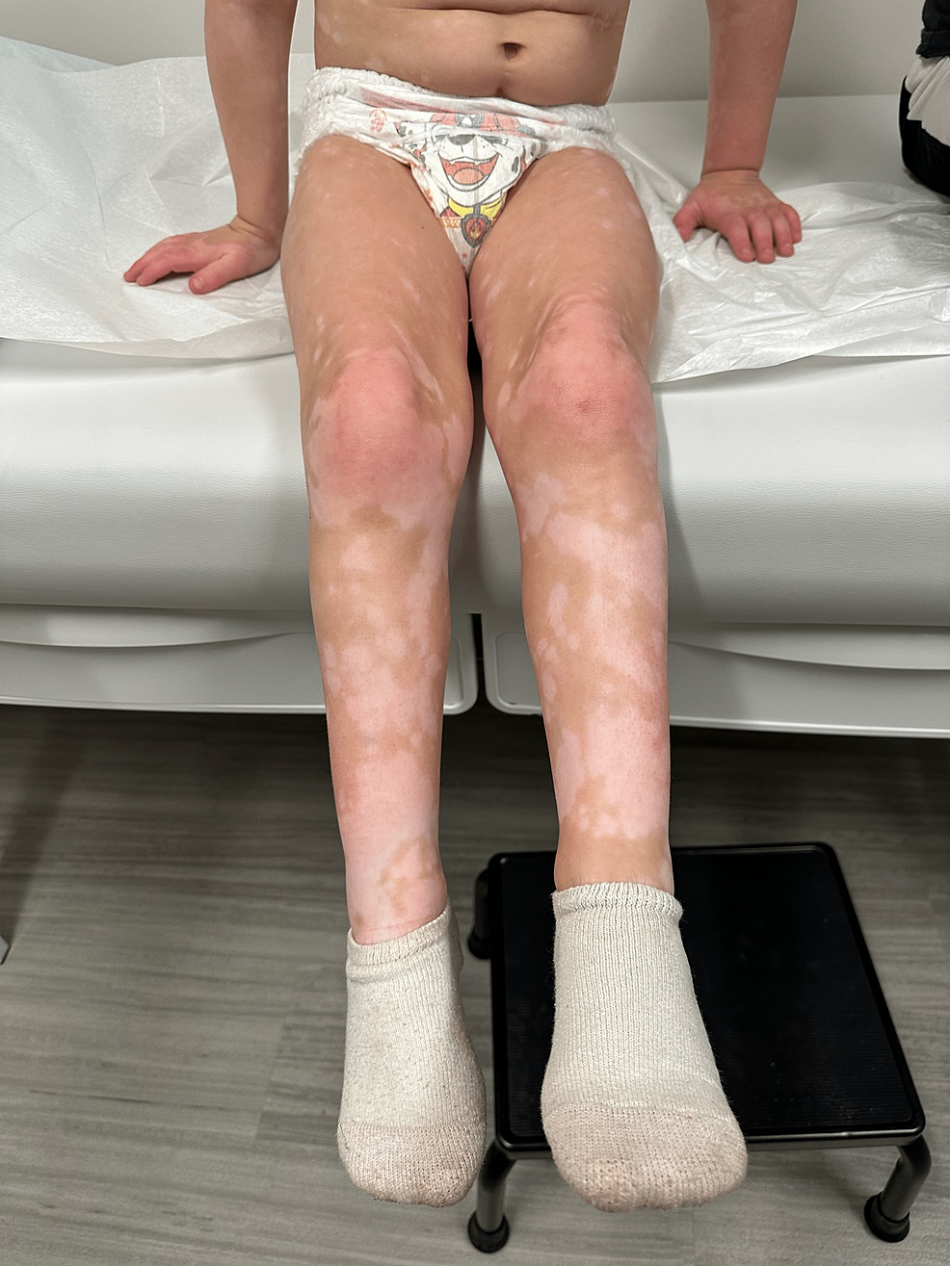
Depigmented macules and patches on the patient's lower extremities at four-month follow-up

**Figure 8:**
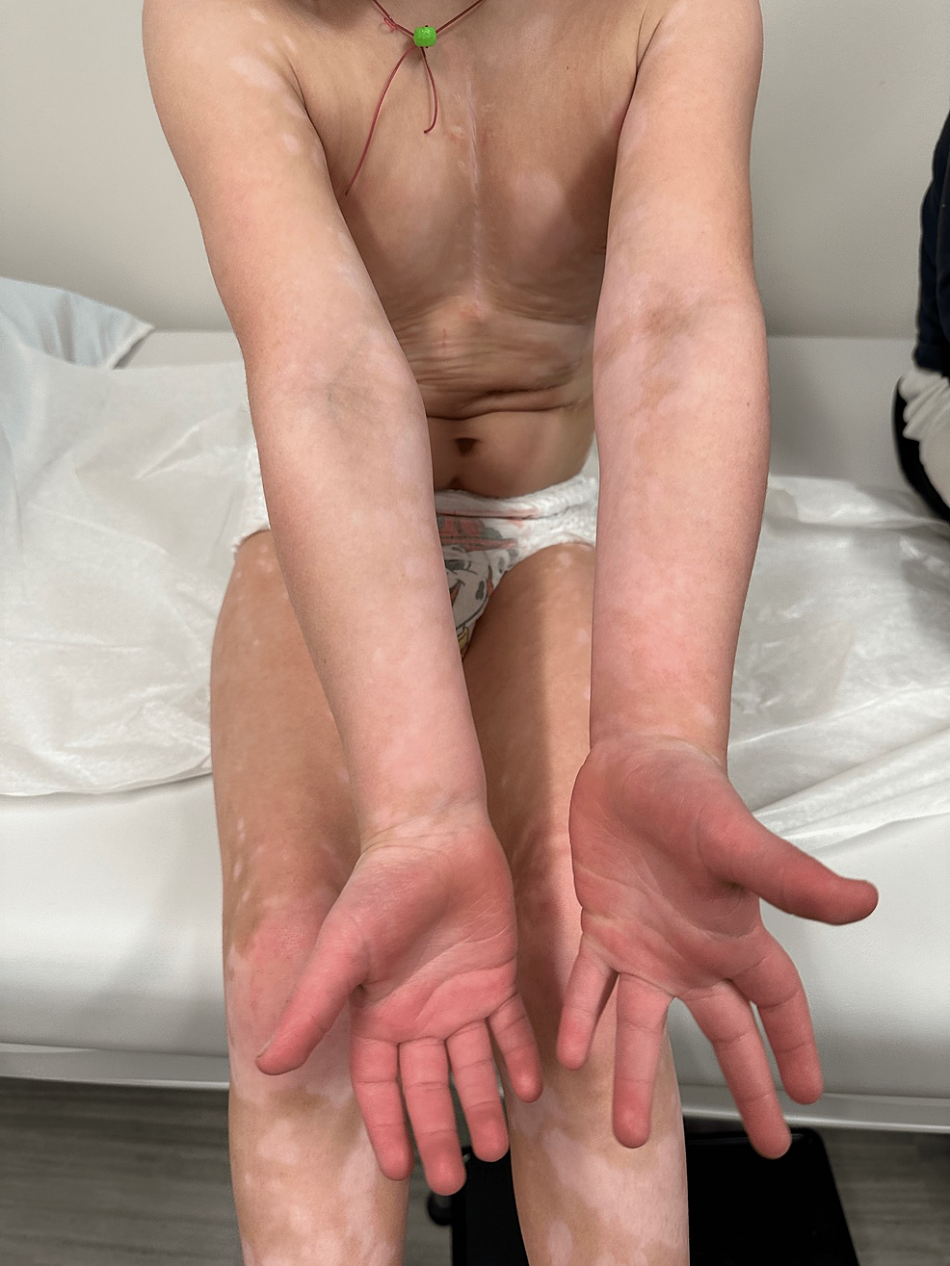
Depigmented macules and patches on the patient's upper extremities at four-month follow-up

At a four-month follow-up visit, there was no improvement in the vitiligo lesions (Figures 6-8). Treatment was changed to topical clobetasol 0.05% ointment applied twice daily every other week with topical tacrolimus 0.03% ointment to be applied twice daily in the weeks clobetasol is not used. 

## Discussion

In 2018, Adam et al. published the diagnostic criteria for KS in collaboration with an international group of experts. This group established that a definite diagnosis of KS can be made in patients with a history of infantile hypotonia, developmental delay, and/or intellectual disability with at least one of the following major criteria: a pathogenic or likely pathogenic variant in KMT2D or lysine-specific demethylase 6 A (KDM6A) or typical dysmorphic facial features [5]. The typical dysmorphic facial features of KS include lateral lower eyelids, arched eyebrows with a sparseness of the lateral sides, long palpebral fissures, short columella, a depressed nasal tip, and prominent ears [1].

While KMT2D is the most common mutation, KDM6A is mutated in about five percent of KS individuals. These genes are involved in a complex of proteins associated with COMPASS, or complex of proteins associated with Set1, which modifies epigenetic marks on histones during chromatin remodeling [2]. The underlying genetic defect remains unknown in approximately 25% of KS patients. Most cases are sporadic; however, autosomal dominant familial occurrence has been reported [1]. The common phenotype produced from these genetic changes includes skeletal anomalies such as brachydactyly and a deformed spinal column and mild to moderate intellectual disability [6]. Other findings that have been observed in patients with KS include congenital heart defects, cleft lip/palate, feeding difficulties, gastric reflux, seizures, urinary tract anomalies, hearing loss, joint hyperlaxity, susceptibility to infections (especially otitis media), and autoimmune diseases [1,4,6].

Autoimmune disorders have been reported with an increased frequency in patients with KS. The most common autoimmune disease reported is ITP [4]. Autoimmune hemolytic anemia, autoimmune thyroiditis, type one diabetes, Crohn’s disease, membranous glomerulonephritis type three, systemic lupus erythematosus, and vitiligo have also been seen in patients with KS [1,2,6]. Vitiligo is reported in one percent of KS cases [3]. In a PubMed and Google Scholar literature search using the phrase “kabuki syndrome and vitiligo”, only 20 reported cases were found in English literature. Of those cases, nine patients were female, 10 were male, and one was unspecified. Most cases of vitiligo in KS were found in children with 10 reported in childhood, four in teenagers, four in adults, and two not specified. Other characteristics seen in these patients include recurrent respiratory tract infections, otitis media, cardiac anomalies, hypogammaglobulinemia, developmental delay, gastrointestinal abnormalities, hypodontia, joint hyperlaxity, hearing loss, cleft palate, renal malformations, and other autoimmune conditions. Four patients had a concurrent autoimmune condition: Hashimoto thyroiditis, autoimmune thyroiditis, primary sclerosing cholangitis, and ITP (Table 2) [1-4,7-13].

**Table 2:** Summary of case reports of patients with Kabuki Syndrome and vitiligo NS = Not stated; ITP = immune thrombocytopenia purpura; # = Number; ^†^Adult is defined as 18 years or older and child as less than 18 years old.

Case #	Reference	Age categorization at onset^†^	Gender	Examination	Concurrent conditions	Other Autoimmune Disease
1 - 9	Margot, et al. [2]	2 children, 4 teenagers, 3 adults	3 females, 6 males	NS	NS	None
10	Di Candia, et al. [7]	Adult	NS	NS	NS	None
11	Stagi, et al. [1]	NS	Male	NS	Respiratory tract infections and otitis media	None
12	Zannoli, et al. [3]	Child	Male	Sharply circumscribed, depigmented macules around the eyes that spread to the hands, feet, arms, legs, and trunk	Palmoplantar dyshidrotic eczema, mental delay, speech delay, hypogammaglobulinemia A and G, behavioral problems, seizures, recurrent respiratory infections, left temporal lobe hypoplasia, and retro cerebellar cysts	None
13	Genevieve, et al. [8]	Child	Female	NS	Ventricular septal defect, neutropenia, hypogammaglobulinemia A and G, chronic diarrhea, persistent hypoglycemic episodes, severe feeding difficulties requiring gastrostomy, and hypodontia	ITP
14	Genevieve, et al. [8]	NS	Male	NS	Pierre Robin sequence, right hip dislocation, bilateral cryptorchidism, bilateral hearing loss, optic nerve coloboma and right microphthalmia, severe scoliosis, right diaphragmatic hernia, atrial septal defect, and pseudarthrosis of the right clavicle	None
15	Ming, et al. [4]	Child	Male	Hypopigmented regions on the fingers that progressed to irregular hypopigmented macules on the legs and trunk	Esotropia, ventricular septal defect, coarctation of the aorta, joint laxity, hypodontia, short stature, and developmental delay	None
16	McGaughran, et al. [9]	Child	Female	Perioral hypopigmentation	Cleft palate and otitis media	None
17	Schrander-Stumpel, et al. [10]	Child	Female	Sharply circumscribed depigmented macules on the scalp, trunk, and lower extremities Poliosis of scalp hair	Somatic and psychomotor retardation, cleft palate, recurrent respiratory infections, and short stature	None
18	Ewart-Toland, et al. [11]	Child	Female	NS	Dysplastic kidneys, coronal synostosis, and hypogammaglobulinemia A	Hashimoto thyroiditis
19	Gurbuz, et al. [12]	Child	Female	Hypopigmented lesions of different sizes on the neck	Sensorineural hearing loss, moderate mental retardation, brachydactyly, joint hyperlaxity, and short stature	Autoimmune thyroiditis
20	Suskind, et al. [13]	Child	Female	Hypopigmented lesions over hands and legs	Developmental delays, coarctation of the aorta, cleft palate, gastrointestinal abnormality requiring Nissen fundoplication, dysplasia of the right hip, and renal dysplasia	Primary Sclerosing Cholangitis
21	Current case	Child	Female	Coalescing depigmented patches on the neck, trunk, and extremities	Developmental delay, imperforate anus with recto-vestibular fistula, gastrointestinal malrotation, gastric reflux, feeding difficulties, pulmonary hypertension, coarctation of the aorta, left aortic arch with aberrant right subclavian artery, mitral stenosis, bicuspid aortic valve, ventricular septal defect, left hydronephrosis, right renal dysplasia, chronic otitis media, bilateral hearing loss, and congenital dislocation of the right hip	None

Vitiligo is commonly treated with topical corticosteroids and calcineurin inhibitors, both of which promote re-pigmentation. Systemic steroids are effective to stabilize an uncontrolled disease. Light therapy can also be used, with narrow-band ultraviolet B (nbUVB) being shown to decrease vitiligo lesions with fewer adverse effects compared to psoralen plus ultraviolet A (PUVA) phototherapy. NbUVB phototherapy can also be used in combination with topical therapies for an additive effect [14]. The downfall of using these medications long-term is their potential side effects. Systemic steroids have a vast, undesirable side effect profile including adrenal suppression, hypertension, dyslipidemia, electrolyte disturbances, arrhythmias, psychiatric disturbances, cushingoid features, dyspepsia, gastric ulcers, hyperglycemia, increased risk for infections, osteoporosis, osteonecrosis, and cataracts. Topical steroids allow for fewer side effects; however, they still endorse undesirable reactions such as skin atrophy and striae, acneiform eruptions, folliculitis, hypopigmentation, and increased risk for secondary skin infections. Adverse effects of topical calcineurin inhibitors include a burning sensation of the skin, pruritis, erythema, and an increased risk of secondary skin infections [15]. Studies utilizing PUVA have demonstrated an increased risk of skin cancers, while nbUVB has not been shown to cause an increased risk of skin cancer. While nbUVB may not increase carcinogenesis, short-term adverse effects include erythema, skin dryness, blistering, pruritis, and increased frequency of recurrent herpes simplex [16].

Janus kinase (JAK) inhibitors have recently been researched in the treatment of vitiligo. JAK inhibitors target the JAK/signal transducer and activator of transcription (STAT)-1 signaling pathway. The JAK/STAT pathway is involved in interferon-gamma and chemokine secretion by keratinocytes which recruit CXCR3+ CD8+ T cells that promote melanocyte detachment and apoptosis. Targeting this pathway has been shown to be effective in blocking interferon-gamma signaling, contributing to re-pigmentation of vitiligo lesions. Ruxolitinib is a selective JAK1 and JAK2 inhibitor. Oral ruxolitinib was first approved in 2011 for the treatment of polycythemia vera, essential thrombocytopenia, and myelofibrosis. Systemic effects of JAK inhibitors include an increased incidence of malignancy, serious infections, and thrombosis based on data from oral use in rheumatoid arthritis. When applied topically, ruxolitinib has resulted in higher medicinal concentrations in the epidermis and dermis with minimum systemic effects [17]. Results from a phase three, randomized, double-blind study of ruxolitinib cream in the treatment of atopic dermatitis showed that only six percent of the topical cream became bioavailable [18]. Trials using 1.5% topical ruxolitinib applied twice daily in vitiligo patients have demonstrated improvement in vitiligo lesions, including lesions that previously failed topical corticosteroid and calcineurin inhibitors [19]. Ruxolitinib 1.5% cream applied twice daily has been shown to have minimal side effects with the most common being application site pruritis [20]. Currently, topical ruxolitinib is only approved for patients above 12 years of age; more research is needed in patients under 12 years old to determine its effectiveness in young children with vitiligo [19].

## Conclusions

In conclusion, KS is a rare genetic disorder with a variety of clinical manifestations. Autoimmune conditions such as vitiligo are seen more frequently in KS patients. In a patient presenting with vitiligo and dysmorphic facial features, healthcare providers should have a clinical suspicion for KS and perform a thorough review of symptoms. More research is needed on the use of topical JAK inhibitors in children as this medication has minimal systemic adverse effects and has worked in patients that failed standard treatment methods.
